# Depression Risk Prediction for Chinese Microblogs via Deep-Learning Methods: Content Analysis

**DOI:** 10.2196/17958

**Published:** 2020-07-29

**Authors:** Xiaofeng Wang, Shuai Chen, Tao Li, Wanting Li, Yejie Zhou, Jie Zheng, Qingcai Chen, Jun Yan, Buzhou Tang

**Affiliations:** 1 School of Communication Shenzhen University Shenzhen China; 2 Department of Computer Science Harbin Institute of Technology Shenzhen Graduate School Shenzhen China; 3 Yidu Cloud (Beijing) Technology Co Ltd Beijing China

**Keywords:** depression risk prediction, deep learning, pretrained language model, Chinese microblogs

## Abstract

**Background:**

Depression is a serious personal and public mental health problem. Self-reporting is the main method used to diagnose depression and to determine the severity of depression. However, it is not easy to discover patients with depression owing to feelings of shame in disclosing or discussing their mental health conditions with others. Moreover, self-reporting is time-consuming, and usually leads to missing a certain number of cases. Therefore, automatic discovery of patients with depression from other sources such as social media has been attracting increasing attention. Social media, as one of the most important daily communication systems, connects large quantities of people, including individuals with depression, and provides a channel to discover patients with depression. In this study, we investigated deep-learning methods for depression risk prediction using data from Chinese microblogs, which have potential to discover more patients with depression and to trace their mental health conditions.

**Objective:**

The aim of this study was to explore the potential of state-of-the-art deep-learning methods on depression risk prediction from Chinese microblogs.

**Methods:**

Deep-learning methods with pretrained language representation models, including bidirectional encoder representations from transformers (BERT), robustly optimized BERT pretraining approach (RoBERTa), and generalized autoregressive pretraining for language understanding (XLNET), were investigated for depression risk prediction, and were compared with previous methods on a manually annotated benchmark dataset. Depression risk was assessed at four levels from 0 to 3, where 0, 1, 2, and 3 denote no inclination, and mild, moderate, and severe depression risk, respectively. The dataset was collected from the Chinese microblog Weibo. We also compared different deep-learning methods with pretrained language representation models in two settings: (1) publicly released pretrained language representation models, and (2) language representation models further pretrained on a large-scale unlabeled dataset collected from Weibo. Precision, recall, and F1 scores were used as performance evaluation measures.

**Results:**

Among the three deep-learning methods, BERT achieved the best performance with a microaveraged F1 score of 0.856. RoBERTa achieved the best performance with a macroaveraged F1 score of 0.424 on depression risk at levels 1, 2, and 3, which represents a new benchmark result on the dataset. The further pretrained language representation models demonstrated improvement over publicly released prediction models.

**Conclusions:**

We applied deep-learning methods with pretrained language representation models to automatically predict depression risk using data from Chinese microblogs. The experimental results showed that the deep-learning methods performed better than previous methods, and have greater potential to discover patients with depression and to trace their mental health conditions.

## Introduction

### Background

Mental health is an important component of personal well-being and public health as reported by the World Health Organization (WHO) [1]. Anyone—regardless of gender, financial status, and age—may suffer from mental disorders, among which depression remains the most common form [2]. Depression is reported to affect more than 264 million people worldwide according to the WHO’s Comprehensive Mental Health Action Plan 2003-2020 [3], and the number has been quickly increasing in recent years [4]. Among various depressive illnesses, the lifetime prevalence of major depressive disorders is approximately 16%, and evidence suggests that the incidence is increasing [5]. In 1997, the WHO estimated that depression will be the second most debilitating disease by 2020, behind cardiovascular disease [6].

Depression is accompanied by a suite of very negative effects, as it can interfere with a person’s daily life and routine. In the short term, depression may reduce an individual’s enjoyment of life, make them withdraw from their family and friends, and ultimately feel lonely. In the long term, prolonged depression may lead to more serious conditions and illnesses. Fortunately, early recognition and treatment are proven to be helpful for people with depression to reduce the negative impacts of the disorder [7]. Despite broad developments in medical technology, it remains difficult to diagnose depression due to the particularity of mental disorders [8]. Currently, most diagnoses of depressive illness are based on self-reports or self-diagnosis of patients [9,10]. The diagnosis procedures are complex and time-consuming. Moreover, a high proportion of patients with depression cannot be discovered as they do not want to disclose or discuss their mental health conditions with others. Therefore, it is urgent to find methods that can help to discover patients with depression from other channels.

With the development of information technology, social media has become an important part of people’s daily life. More and more people are using social media platforms such as Twitter, Facebook, and Sina Weibo to share their thoughts, feelings, and emotional status. These social media platforms can provide a huge amount of valuable data for research. Some studies based on social media data such as personalized news recommendation [11], public opinion sensing and trend analysis [12], disease transmission trend monitoring [13], and future patient visits prediction [14] have achieved good results. In the case of depression, as social media platforms have become important forums for people with depression to interact with peers within a comfortable emotional distance [15], high numbers of patients with depression tend to gather to share their feelings, emotional status, and treatment procedures. Some researchers have attempted to discover patients with depression from social media, such as by predicting depression risk embedded in text from microblogs. Accumulating evidence shows that the language and emotion posted on social media platforms could indicate depression [3].

In this study, we investigated the use of deep-learning methods for depression risk prediction from data collected in Chinese microblogs. This study represents an extension of the study of Wang et al [16], who presented an annotated dataset of Chinese microblogs for depression risk prediction and compared four machine-learning methods, including the deep-learning method bidirectional encoder representations from transformers (BERT) [17]. Here, we further investigated three deep-learning methods with pretrained language representation models, BERT, robustly optimized BERT pretraining approach (RoBERTa) [18], and generalized autoregressive pretraining for language understanding (XLNET) [19], on the depression dataset and obtained new benchmark results.

### Related Work

In early studies focused on depression detection, most of the methods applied were rule-based and those based on self-reporting or self-diagnosis. For example, Hamilton [20] established a rating scale for depression to help patients with depression evaluate the severity of their depression by themselves according to a self-report. However, these methods always require domain experts to define the rules and are time-consuming. In recent years, with the rapid spread of social media, more and more information about personal daily life is publicly posted on the internet, which can be widely used for health prediction, including depression detection.

Choudhury et al [9] made a major contribution to the field of depression detection from social media by investigating whether social media can be used as a source of information to detect mental illness among individuals as well as within a population. Following this study, several researchers annotated some corpora for automatic depression detection, including depression level prediction. For example, Glen et al [21] constructed an annotated corpus composed of 1746 users collected from Twitter for depression detection. In the corpus, the users were divided into three groups: depression users, posttraumatic stress disorder (PTSD) users, and control users. This corpus was used as the dataset of the Computational Linguistics and Clinical Psychology (CLPsych) shared task in 2015 [22] to predict PTSD users from the control group, users with depression from the control group, and users with depression among users with PTSD. The system that ranked first in the CLPsych 2015 shared task was a combination system composed of 16 support vector machine (SVM)-based subsystems based on features derived using supervised linear discriminant analysis [23], supervised Anchor (for topic modeling), and lexical term frequency-inverse document frequency [24]. Cacheda et al [25] presented a social network analysis and random forest algorithm to detect early depression. Ricard et al [26] trained an elastic-net regularized linear regression model on Instagram post captions and comments to detect depression. The features used in the linear regression model included multiple sentiment scores, emoji sentiment analysis results, and metavariables such as the number of “likes” and average comment length. Lin et al [27] proposed a deep neural network model to detect users’ psychological stress by incorporating two different types of user-scope attributes, and evaluated the model on four different datasets from major microblog platforms, including Sina Weibo, Tencent Weibo, and Twitter. Most of these studies focused on user-level depression detection, as summarized by Wongkoblap et al [28], and the machine-learning methods used in these studies included SVM, logistic regression, decision trees [29-32], random forest [33,34], naive Bayes [35,36], K-nearest neighbor, maximum entropy [37], neural network, and deep-learning neural network.

To analyze social media at a fine-granularity level and track the mental health conditions of patients with depression, some researchers attempted to detect depression at the tweet level. Jamil et al [38] constructed two types of datasets from Twitter for depression detection: one annotated at the tweet level consisting of 8753 tweets and the other annotated at the user level consisting of 160 users. The SVM-based system developed on these two datasets performed well at the user level, but not very well at the tweet level. Wang et al [16] annotated a dataset from Sina Weibo at the microblog level (equivalent to the tweet level), in which each microblog was labeled with a depression risk ranging from 0 to 3. They compared four machine-learning methods on this dataset, including SVM, convolutional neural network (CNN), long short-term memory network (LSTM), and BERT. The three deep-learning methods (ie, CNN, LSTM, and BERT) significantly outperformed SVM, and BERT showed the best performance among them.

During the last 2 or 3 years, pretrained language representation models such as BERT, RoBERTa, and XLNET have shown significant performance gains in many natural language processing tasks such as text classification, question answering, and others [39]. However, to the best of our knowledge, deep-learning methods with pretrained language representation models have not yet been applied to depression risk prediction.

## Methods

### Dataset

In this study, we use the dataset provided by Wang et al [16], which was collected from the Chinese social media platform Sina Weibo. In this dataset, 13,993 microblogs were annotated with depression risk assessed at four levels from 0 to 3, where 0 indicates no inclination to depression, or only some common pressures such as work, study, and family issues; 1 indicates mild depression, denoting that users express despair with life but do not mention suicide or self-harm; 2 indicates moderate depression, which denotes that users mention suicide or self-harm without stating a specific time or place; and 3 indicates severe depression, which denotes that users mention suicide or self-harm with a specific time or place. A total of 11,835 microblogs were annotated as 0, 1379 microblogs were annotated as 1, 650 microblogs were annotated as 2, and the remaining 129 microblogs were annotated as 3. The distribution of microblogs at different levels was imbalanced. Table 1 provides examples of the different depression levels. Following Wang et al [16], we split the dataset into two parts: a training set of 11,194 microblogs and a test set of 2799 microblogs, as shown in Table 2.

**Table 1:** Examples of different depression risk levels in the dataset.

Depression risk level	Microblog
3	Weibo: 不出意外的话，我打算死在今年 。 Barring accidents, I plan to commit suicide this year.
2	Weibo: 我一直策划着如何自杀，可是放不下的太多了。 I have been planning to commit suicide, but I cannot let go of too many things.
1	Weibo: 如果我累，真的离开了。 If I’m tired, I will leave.
0	Weibo: 吃了个早餐应该能维持今天。 The breakfast I ate should be able to support me today.

**Table 2:** Dataset statistics.

Depression level	Training set (n)	Test set (n)
3	103	26
2	520	130
1	1103	276
0	9468	2367
All	11,194	2799

### Deep-Learning Methods Based on Pretrained Language Representation Models

#### BERT

BERT is a language representation model designed to pretrain deep bidirectional representations from unlabeled text by jointly conditioning on both the left and right context in all layers [17]. It uses the transformer architecture to capture long-distance dependences in sentences. During pretraining, BERT optimizes the masked language model (MLM) and the next sentence prediction (NSP) task jointly on large-scale unlabeled text. To implement NSP, BERT adds the token [CLS] at the beginning of every sequence. The final hidden state corresponding to the token [CLS] is then used as the aggregate sequence representation for downstream tasks. When the language representation model is pretrained, it can be subsequently fine-tuned for downstream tasks using the labeled data of downstream tasks. BERT achieved better performance on several natural language processing tasks in 2018 [17]. In the present study, depression risk prediction was formalized as a classification task; therefore, we simply needed to feed the representation of token [CLS] into an output layer (a fully connected layer) and then fine-tune the whole network.

#### RoBERTa

RoBERTa is an optimized replication version of BERT [18]. Compared with BERT, RoBERTa offers the following four improvements during training: (1) training the model for a longer period with larger batches over more data; (2) removing the NSP task; (3) training on longer sequences; and (4) dynamically changing the masking pattern applied to the training data. Based on these improvements, RoBERTa has achieved new state-of-the-art results on many tasks compared with BERT [18].

#### XLNET

XLNET is a generalized autoregressive method that takes advantage of both autoregressive language modeling and autoencoding while avoiding their limitations [19]. As BERT and its variants (eg, RoBERTa) neglect the dependency between the masked positions and suffer from a pretrain-finetune discrepancy, XLNET adopts a permutation language model instead of MLM to solve the discrepancy problem. For downstream tasks, the fine-tuning procedure of XLNET is similar to that of BERT and RoBERTa.

### Experiments

#### Experimental Setup

We investigated the different deep-learning methods with pretrained language representation models in two settings: (1) publicly released pretrained language representation models and (2) language representation models further pretrained on a large-scale unlabeled dataset collected from Weibo based on (1). The hyperparameters for BERT, RoBERTa, and XLNET for depression risk prediction are listed in Table 3. These hyperparameters were obtained by crossvalidation.

**Table 3:** Hyperparameters for the deep-learning methods.

Parameter	BERT^a^	RoBERTa^b^	XLNET^c^
Learning rate	1e-5	1e-5	2e-5
Training steps	7000	7000	7000
Maximum length	128	128	128
Batch size	16	16	16
Warm-up steps	700	700	700
Dropout rate	0.3	0.3	0.3

^a^BERT: bidirectional encoder representations from transformers.

^b^RoBERTa: robustly optimized bidirectional encoder representations from transformers pretraining approach.

^c^XLNET: generalized autoregressive pretraining for language understanding.

#### In-Domain Pretraining

For in-domain pretraining (IDP), we started from the public released pretrained BERT model [40], RoBERTa model [41], and XLNET model [42], and further pretrained them on the same unlabeled Weibo corpus as used by Wang et al [16]. The unlabeled corpus contains about 300,000 microblogs. The hyperparameters used during further IDP are listed in Table 4. These hyperparameters were optimized by crossvalidation.

**Table 4:** Hyperparameters during further in-domain pretraining for the deep-learning methods.

Parameter	BERT^a^	RoBERTa^b^	XLNET^c^
Learning rate	2e-5	2e-5	2e-5
Training steps	100,000	100,000	100,000
Maximum length	256	256	256
Batch size	16	16	16
Warm-up steps	10,000	10,000	10,000

^a^BERT: bidirectional encoder representations from transformers.

^b^RoBERTa: robustly optimized bidirectional encoder representations from transformers pretraining approach.

^c^XLNET: generalized autoregressive pretraining for language understanding.

#### Evaluation Criteria

Micro/macro precision, recall, and the F1 score were used to evaluate the performance of the different deep-learning methods.

## Results

Table 5 shows the performance of deep-learning methods with different language representation models. For each deep-learning method, the addition of a pretrained language representation model brought improvement over the publicly released language representation model. Among the three methods, BERT showed the best performance, with the highest microF1 score of 0.856 (BERT_IDP). The microF1 score difference between any two of the three methods was around 1%-2%, which is not satisfactory. Compared with CNN and LSTM, BERT, RoBERTa, and XLNET showed a great advantage.

Almost all of the deep-learning methods performed the best on level 0 and performed the worst on level 3, which may be caused by data imbalance. For all depression risk levels except for level 0, the deep-learning methods showed different performance rankings. On level 1, RoBERTa_IDP performed the best with an F1 score of 0.422, whereas on level 2, XLNET_IDP achieved the best F1 score of 0.493, and on level 3, XLNET achieved the best F1 score of 0.445.

As the aim of this study was to discover potential patients with depression, we were more interested in microblogs at levels 1, 2, and 3. Therefore, it is more meaningful to report macro precision, recall, and F1 scores on these three levels, which are shown in Table 6, in which the highest values in each column are in italics. The advantage of RoBERTa_IDP for microblog-level depression detection can be clearly seen. The confusion matrices of BERT_IDP, RoBERTa_IDP, and XLNET_IDP are shown in Table 7.

**Table 5:** Performance of deep-learning methods with different language representation models.

Model	Level-0				Level-1				Level-2				Level-3				MicroF1
	P^a^	R^b^	F1	P		R	F1	P		R	F1	P		R	F1		
CNN^c^ [16]	0.908	0.940	0.924	0.380		0.236	0.291	0.351		0.415	0.380	0.250		0.231	0.240	0.841	
LSTM^d^ [16]	0.896	0.936	0.916	0.294		0.288	0.257	0.324		0.262	0.289	0.714		0.192	0.303	0.832	
BERT^e^ [16]	0.942	0.894	0.917	0.323		0.502	0.393	0.468		0.489	0.478	0.574		0.152	0.240	0.834	
BERT_IDP^f^ [16]	0.929	0.938	*0.934* ^g^	0.394		0.446	0.418	0.568		0.385	0.459	0.667		0.231	0.343	*0.856*	
RoBERTa^h^	0.931	0.920	0.925	0.355		0.464	0.402	0.556		0.385	0.455	0.600		0.231	0.333	0.843	
RoBERTa_IDP	0.933	0.920	0.926	0.371		0.489	*0.422*	0.578		0.400	0.473	0.636		0.269	0.333	0.847	
XLNET^i^	0.908	0.948	0.927	0.358		0.273	0.309	0.484		0.353	0.408	0.530		0.384	*0.445*	0.848	
XLNET_IDP	0.933	0.920	0.926	0.361		0.471	0.409	0.577		0.431	*0.493*	0.625		0.192	0.294	0.846	

^a^P: precision.

^b^R: recall.

^c^CNN: convolutional neural network.

^d^LSTM: long short-term memory network.

^e^BERT: bidirectional encoder representations from transformers.

^f^_IDP: The model is further trained on the in-domain unlabeled corpus.

^g^Highest F1 values are indicated in italics.

^h^RoBERTa: robustly optimized bidirectional encoder representations from transformers pretraining approach.

^i^XLNET: generalized autoregressive pretraining for language understanding.

**Table 6:** Performance of deep-learning methods with different language representation models on level 1, 2 and 3.

Model	Macro-F1	Macro-P^a^	Macro-R^b^
BERT^c^ [16]	0.370	0.455	0.381
BERT_IDP^d^ [16]	0.406	*0.543* ^e^	0.354
RoBERTa^f^	0.396	0.503	0.360
RoBERTa_IDP	*0.424*	0.528	*0.386*
XLNET^g^	0.387	0.457	0.336
XLNET_IDP	0.398	0.521	0.364

^a^P: precision.

^b^R: recall.

^c^BERT: bidirectional encoder representations from transformers.

^d^_IDP: The model is further trained on the in-domain unlabeled corpus.

^e^Highest F1 values are indicated in italics.

^f^RoBERTa: robustly optimized bidirectional encoder representations from transformers pretraining approach.

^g^XLNET: generalized autoregressive pretraining for language understanding.

**Table 7:** Confusion matrix of the deep-learning methods with in-domain training.

Gold-standard method		Prediction method Level-0	Prediction method Level-1	Prediction method Level-2	Prediction method Level-3
**BERT_IDP^a^**					
	Level-0	2221	131	14	1
	Level-1	137	123	16	0
	Level-2	26	52	50	2
	Level-3	6	6	8	6
**RoBERTa_IDP^b^**					
	Level-0	2177	176	13	1
	Level-1	128	135	15	0
	Level-2	26	47	52	3
	Level-3	3	6	10	7
**XLNET_IDP^c^**					
	Level-0	2177	176	13	1
	Level-1	128	130	18	0
	Level-2	26	46	56	2
	Level-3	3	8	10	5

^e^BERT_IDP: bidirectional encoder representations from transformers further trained on the in-domain unlabeled corpus.

^b^RoBERTa_IDP: robustly optimized bidirectional encoder representations from transformers pretraining approach further trained on the in-domain unlabeled corpus.

^c^XLNET_IDP: generalized autoregressive pretraining for language understanding further trained on the in-domain unlabeled corpus.

## Discussion

### Principal Findings

In this study, we have applied three deep-learning methods with pretrained language representation models to predict the depression risk based on data from Chinese microblogs, which is recognized as a text classification task. The deep-learning methods achieved the highest macroaveraged F1 score of 0.424 on the three levels of depression of concern, which represents a new state-of-the-art result from the dataset used by Wang et al [16]. These results indicate the potential for tracing mental health conditions of depression patients from microblogs. We also investigated the effect of pretraining language representation models in different settings. These experiments showed that further applying pretrained language representation models on a large-scale unlabeled in-domain corpus leads to better performance, which is easily interpretable.

Error analysis on the deep-learning methods showed that several errors often occur between level 0 and level 1. As shown in the confusion matrix in Table 7, among all samples predicted incorrectly by RoBERTa_IDP, 128 gold-standard samples at level 1 were predicted as level 0 and 176 gold-standard samples at level 0 were predicted as level 1. This type of error accounted for about 70% of all errors. The main reason for this phenomenon is that there are many ambiguous words in Chinese microblogs, which are difficult to be distinguished independently. These ambiguous words also occurred very frequently in microblogs of high depression risk levels. For example, in microblog “我已经放下了亲情、友情，都已经和解了，可以安心上路了(I have let go of my family and friendships, and have reconciled with them. Now, I can go on my way with ease),” “上路” is an ambiguous word. In Chinese, this word not only means “going on one’s way” but also has the meaning of passing away. Other examples include ”解脱 (extricate)” in “啥时候能够解脱呢？有点期待 (When can I extricate myself from the tough world? I am looking forward to it),” and “黑(black)” in “我看到的世界都是黑的只剩下一片黑 (The world I see is black, only black).” These words are not related to depression risk in most common contexts. However, in the contexts mentioned above, these words indicate the despair of patients in life. Since these words appeared infrequently in the entire depression dataset, it was very difficult for the deep-learning models to learn the multiple meanings of these ambiguous words. From the confusion matrix, we can see that RoBERTa_IDP could correctly classify more samples at a high level than the previous BERT model. This suggests that our new methods can handle these types of errors better than previous methods. For these types of errors, there may be two possible solutions: one is to import more samples containing these ambiguous words to help the models learn the multiple meanings of these words, and the other is to import more of the context from the same user to help the models make a correct prediction.

In the future, there may be three directions for further improvement. First, we will expand the current dataset to cover as many multiple meanings of ambiguous words as possible. Second, we will attempt to use user-level context to improve microblog-level depression risk prediction. Third, we will try to add medical knowledge regarding depression into the deep-learning methods.

### Conclusion

Depression is one of the most harmful mental disorders worldwide. The diagnosis of depression is quite complex and time-consuming. Predicting depression risk automatically is very important and meaningful. In this study, we have focused on the potential of deep-learning methods with pretrained language representation models for depression risk prediction from Chinese microblogs. The experimental results on a benchmark dataset showed that the proposed methods performed well for this task. The main contribution of this study to depression health care is to help discover potential patients with depression from social media quickly. This could help doctors or psychologists to concentrate on providing help for these potential patients with a high depression level.

## Abbreviations

BERT: bidirectional encoder representations from transformers
CLPsych: Computational Linguistics and Clinical Psychology
CNN: convolutional neural network
IDP: in-domain pretraining
LSTM: long short-term memory network
MLM: masked language model
NSP: next sentence prediction
PTSD: posttraumatic stress disorder
RoBERTa: robustly optimized bidirectional encoder representations from transformers pretraining approach
SVM: support vector machine
WHO: World Health Organization
XLNET: generalized autoregressive pretraining for language understanding
